# Treatment of Co-Occurring Alcohol and Other Drug Use Disorders

**Published:** 2008

**Authors:** Albert J. Arias, Henry R. Kranzler

**Keywords:** Alcohol and other drug use disorder (AODUD), dual addiction, co-morbid AOD dependence, treatment methods, psychosocial treatment method, behavior therapy, pharmacotherapy, combined treatment, literature review

## Abstract

Drug use disorders (DUDs) frequently co-occur with alcohol use disorders, affecting approximately 1.1 percent of the U.S. population. Compared with alcohol use disorders or DUDs alone, co-occurring disorders are associated with a greater severity of substance dependence; co-occurring psychiatric disorders also are common in this patient population. Many effective medications and behavioral treatments are available to treat alcohol dependence and drug dependence when these occur independent of one another. There is a paucity of research, however, specifically focused on the treatment of persons with co-occurring alcohol and other DUDs (AODUDs). The evidence to date on treating this patient population suggests that combining some of the behavioral and pharmacologic treatments that are effective in treating either drug or alcohol use disorders alone may be useful in the AODUD population as well.

An estimated 1.1 percent of the U.S. population has an alcohol use disorder with a co-occurring drug use disorder (DUD) (Stinson et al. 2005). This type of co-morbidity is sometimes referred to as homotypic co-morbidity or dual dependence (Stinson et al. 2005). To be consistent with the theme of this issue, this article refers to people with this combination of disorders as having alcohol and other drug (AOD) use disorders (AODUDs). Many people with alcohol use disorders use other substances at some point in their lives. This article focuses on the following AOD combinations: alcohol and cocaine, alcohol and cannabis, opioids and cocaine, and alcohol and cocaine with methadone maintenance. The drug that most commonly is combined with AODs is nicotine, which, because it was thoroughly discussed in a recent issue of *Alcohol Research & Health* (Volume 29, Number 3), is not discussed here.

After a brief discussion of assessment, placement, and treatment matching, this article reviews the literature on evidence-based pharmacologic and behavioral treatment strategies for AOD dependence. It also presents evidence for using specific treatments for AODUDs and provides recommendations on how to implement these treatments.

## Assessment, Placement, and Treatment Matching

In general, patients with AODUDs have a greater severity of substance dependence than patients with only an alcohol use disorder or a DUD. People with AODUDs are at least as likely to have co-occurring psychiatric disorders as those who have only DUDs and are more likely to have such disorders than those with only alcohol use disorders (Stinson et al. 2005). In addition, people with AODUDs are more likely than those with either drug or alcohol use disorders alone to seek treatment (Stinson et al. 2005). Thus, patients with AODUDs are perhaps best evaluated for treatment planning by a practitioner with specialized expertise in addictive disorders. Although many factors dictate the initial placement and treatment of the AODUD patient (e.g., co-occurring pregnancy or the need for medical detoxification), general guidelines are available. The American Society of Addiction Medicine (ASAM) has published guidelines for placement and treatment matching (*ASAM Patient Placement Criteria for the Treatment of Substance-Related Disorders 2001*). The American Psychiatric Association (APA) also has published guidelines for the treatment of substance use disorders (SUDs), which cover the issue of placement into various treatment settings (Kleber et al. 2007). Treatment that integrates AODUDs and psychiatric care probably is optimal for most AODUD patients, particularly those with greater severity of psychiatric co-morbidity (Drake et al. 2004). Effective integrated, dual-diagnosis programs emphasize the combination of multiple treatment modalities delivered in a format that acknowledges the limitations and nature of co-occurring psychiatric illness and is delivered by a staff skilled in the treatment of both addictive and psychiatric disorders.

## Evidence-Based Treatments for Individual SUDs

Although a complete review of the behavioral and pharmacologic treatment literature for specific addictive disorders is beyond the scope of this article, this section provides a brief summary of the major treatment modalities that currently are in use. In general, an approach that combines behavioral and pharmacologic treatments is optimal for most patients. However, recent findings from studies of the pharmacotherapy of alcohol dependence have shown that some patients may do well when medication is combined with a minimal behavioral approach focusing on medication adherence (Anton et al. 2006*b*; Johnson et al. 2003, 2007). In addition, among alcohol-dependent patients, those with a goal of abstinence from alcohol likely have better treatment outcomes. However, because some patients do not subscribe to such a goal, it often is necessary to negotiate a “harm reduction” approach with them, with the option of modifying the goal if their efforts to reduce their drinking substantially are not successful (Mason et al. 2006). In drug dependence treatment, behavioral therapies often are considered primary and medications secondary, except in the case of patients receiving opioid agonist^1^ maintenance therapy.

### Behavioral Therapies

Table 1 lists the research-based behavioral therapies for different SUDs, with a general description of the level of research evidence supporting each of the treatments (McCaul and Petry 2003). Because people with substance dependence often are ambivalent about changing their behavior, some experts consider motivational enhancement therapy (MET) to be an essential element of addictions treatment, although the evidence supporting its use may be strongest in the treatment of alcohol use disorders (Carroll and Onken 2005; McCaul and Petry 2003). MET aims to engage in treatment patients who are resistant to behavioral change and may be the most acceptable therapeutic approach when patients are new to treatment for AODUDs. MET can help to build a working alliance between the patient and practitioner and provide a foundation on which other useful therapies, including medications, may be added. Cognitive-behavioral therapy (CBT) and MET are effective in the treatment of cannabis dependence (Nordstrom and Levin 2007). Contingency management^2^ interventions also have proven to be effective in treating SUDs (Peirce et al. 2006; Petry and Martin 2002; Prendergast et al. 2006), including reducing both drug use and drinking in alcohol-dependent patients (Petry et al. 2000). In addition to the therapies shown in table 1, an intensive outreach counseling program may be helpful in reducing illicit drug use and returning to treatment patients who drop out from methadone maintenance (Zanis et al. 1996).

A variety of behavioral approaches have shown efficacy in the treatment of alcohol use disorders (see table 1). BRENDA, which is an acronym for Biopsychosocial, Report, Empathy, Needs, Direct advice, and Assessment, has been used to provide a flexible, and practical, client-centered therapy for use in conjunction with pharmacotherapy (Garbutt et al. 2005). BRENDA, like MET, is a client-centered approach that, by building a working alliance with the patient, can act as foundation for other treatments (Pettinati et al. 2000*b*). BRENDA may enhance medication adherence in the treatment of alcohol dependence (Pettinati et al. 2000*b*).

NIAAA also has published several behavioral treatment guides and manuals (http://www.niaaa.nih.gov/Publications/EducationTrainingMaterials/guide.htm).

Volume 1 of the *Project COMBINE* monograph series (Miller 2004), which describes combined behavioral intervention (CBI), provides detailed guidelines for a state-of-the-art counseling approach to the treatment of alcohol dependence that combines elements of CBT and MET.

Volume 2 (Pettinati et al. 2004) describes medication management (MM), an intervention that was used in the COMBINE study (Anton et al. 2006) as a minimal supportive approach to accompany medication therapy in alcohol-dependent patients. In this study, treatment with the opioid receptor antagonist^3^ naltrexone accompanied by MM was efficacious in achieving successful drinking outcomes. However, combining elements of multiple behavioral therapies (e.g., combining motivational interventions with CBT and clinical management) may be the most effective approach to the treatment of SUDs (Carroll 2005).

### Pharmacotherapies

Table 2 summarizes the current research findings on the efficacy of pharmacologic treatments for SUDs. This section will review the relevant findings on treatments for alcohol, cocaine, opioid, and cannabis dependence.

#### Alcohol Dependence

Despite progress in pharmacotherapy research, medications approved to treat alcohol dependence are underutilized (Mark et al. 2003).

##### Disulfiram

This medication causes flushing, nausea, nervousness, and other unpleasant physiologic effects when combined with alcohol. It was approved by the U.S. Food and Drug Administration (FDA) in 1949, making it the first medication approved for treating alcohol dependence. Disulfiram was approved before implementation of the FDA requirement that a medication have demonstrated efficacy as well as safety. In a multicenter trial that included more than 600 male veterans, disulfiram failed to increase the likelihood of abstinence during a 1-year treatment period (Fuller et al. 1986). However, among individuals who drank, a 250-mg dose of disulfiram reduced drinking days relative to a 1-mg dose of disulfiram or a placebo. Note though, the potentially serious adverse effects produced by the medication when combined with alcohol argue for its use in abstinence-oriented treatment rather than for harm reduction.

Patients who are likely to respond well to disulfiram treatment are older; have a longer drinking history; greater social stability, impulsivity, and motivation for recovery; attend Alcoholics Anonymous meetings; and are cognitively intact (Fuller and Gordis 2004; Suh et al. 2006). When disulfiram ingestion in a clinical trial was supervised by an individual designated by the patient, the drug was shown to increase abstinent days and decrease overall drinking relative to a placebo (Chick et al. 1992). Based on these and other findings, the efficacy of disulfiram may depend upon supervised administration of the drug (Hughes and Cook 1997; Suh et al. 2006).

##### Naltrexone

Several meta-analyses support the efficacy of oral naltrexone (at a daily dosage of 50 mg) in the treatment of alcohol dependence (see Spanagel and Zieglgansberger 1997 and table 3 for the putative mechanism of action of the medication). Naltrexone has been shown most consistently to be effective in reducing the risk of relapse to heavy drinking, with less consistent evidence that it reduces the percentage of drinking days or increases the likelihood of total abstinence (Bouza et al. 2004; Srisurapanont and Jarusuraisin 2005). One long-acting formulation of naltrexone has been shown to be effective in reducing heavy drinking among alcohol-dependent patients and may offer enhanced adherence, at least early in treatment, which can be a critical time in the process of recovery for AODUD patients (Garbutt et al. 2005). Based on the findings from that study, the FDA approved long-acting naltrexone for the treatment of alcohol dependence. A second injectable formulation of naltrexone reduced the number of drinking days and increased the likelihood of total abstinence compared with a placebo injection (Kranzler et al. 2004).

##### Acamprosate

Acamprosate is an amino acid derivative that affects the brain signaling molecules (i.e., neurotransmitters) γ-aminobutyric acid (GABA) and glutamate (Spanagel and Zieglgansberger 1997). The mechanism of action of acamprosate (Johnson 2008; Littleton and Zieglgansberger 2003; Spanagel and Zieglgansberger 1997) is shown in table 3.

Meta-analyses of European clinical trials have shown that acamprosate nearly doubles the likelihood of abstinence and reduces the risk of heavy drinking (Bouza et al. 2004; Chick et al. 2003; Mann et al. 2004). Based on these studies, later age of onset of alcohol dependence, the absence of a family history of alcohol dependence, and the presence of physiologic dependence and higher levels of anxiety are associated with a beneficial response to acamprosate (Johnson et al. 2007; Verheul et al. 2005). Based on three pivotal trials from Europe, the FDA approved acamprosate to treat alcohol dependence (Kranzler and Gage 2008). However, two recent studies (Anton et al. 2006*b*; Mason et al. 2006) of acamprosate conducted in the United States show the drug to be no better than placebo, when a standard, intent-to-treat analysis^4^ was used.

Two studies (Kiefer et al. 2003; Anton et al. 2006*b*) of naltrexone plus acamprosate versus either agent alone for the treatment of alcohol dependence did not show a clear advantage of the combination. Kiefer and colleagues (2003) found that the combination treatment was superior to acamprosate alone but was not better than naltrexone alone. In the COMBINE study (Anton et al. 2006*b*), treatment with the combination of naltrexone and acamprosate was no better than placebo treatment.

##### Anticonvulsant Medications

Topiramate is the best studied of the anticonvulsant medications that have been evaluated for the treatment of alcohol dependence. It has been shown to be effective in reducing a variety of drinking outcomes among alcohol-dependent patients (see table 3 for mechanism of action) in both a single-site study (Johnson et al. 2003) and in a randomized, placebo-controlled, double-blind, multicenter trial (Johnson et al. 2007). Studies of other anticonvulsants, including carbamazepine and valproate, also have shown some evidence of efficacy for the treatment of alcohol dependence and may be especially useful in patients with co-occurring bipolar disorders (Brady et al. 2002; Mueller et al. 1997; Salloum et al. 2005).

##### Other Medications

A thorough discussion of serotonergic medications (e.g., selective serotonin reuptake inhibitors [SSRIs], ondansetron) and other medications (e.g., atypical antipsychotics, lithium) is beyond the scope of this review. Currently, there is not sufficient evidence to recommend the use of these agents for the treatment of primary SUDs. Ongoing research may clearly define a role for such medications in treating SUDs, particularly in certain subgroups of alcohol-dependent patients and those with co-occurring psychiatric illness (Anton et al. 2006*a*; Brown et al. 2005; Guardia et al. 2004; Kampman et al. 2007; Monnelly et al. 2004; Johnson et al. 2000; Kranzler et al. 1996, 2006; Kranzler and Ciraulo 2005; Pettinati et al. 2000*a*). Lithium does not appear efficacious for the treatment of alcohol dependence and has not been adequately tested among patients with co-morbid alcohol dependence and bipolar disorder (see Cerullo and Strakowski 2007 and Frye and Salloum 2006 for reviews). In a recent randomized, placebo-controlled clinical trial (Addolorato et al. 2007), baclofen, a GABA_B_ receptor agonist, was shown to be safe and effective for the treatment of alcohol dependence. However, given the possibility of misuse and other complications from baclofen, which have been noted in the medical literature (e.g., withdrawal, psychosis, and delirium), more research with this medication is needed before it can be recommended as a safe and effective treatment for SUDs (Auger and Potash 2005; May 1983; McDonald et al. 2001; Perry et al. 1998).

#### Cocaine Dependence

Although many medications have been evaluated to treat cocaine dependence, few agents have shown efficacy. Currently, disulfiram, tiagabine, topiramate, and modafinil are the most promising of the available treatments (Karila et al. 2007; Vocci and Elkashef 2005).

##### Disulfiram

Some of the initial research with disulfiram was conducted in study participants with alcohol and cocaine dependence and is discussed below. Disulfiram probably reduces cocaine use in cocaine-dependent individuals by producing an aversive reaction to the drug and also may reduce the euphoria produced by the drug, although some studies note an increase in subjective “highs” (see table 3 for more details on the putative mechanism of action) (Baker et al. 2007; Sofuoglu and Kosten 2005; Vocci and Elkashef 2005). Disulfiram also may delay the high from cocaine, decreasing its reinforcement value. Combined with associated increases in plasma concentrations of cocaine, patients may experience greater nervousness and increased cardiovascular strain, which are aversive (Hameedi et al. 1995; McCance-Katz et al. 1998). However, similar to what is seen in the treatment of alcohol dependence with disulfiram, adherence limits the utility of this medication in the treatment of cocaine dependence. Practical approaches to dealing with this problem include making access to a desired treatment (e.g., methadone for co-occurring opioid dependence) contingent on adherence or using social reinforcers (e.g., an agreement or contract between patient and spouse requiring adherence).

##### Tiagabine^5^

A GABA reuptake inhibitor, tiagabine has yielded mixed results for the treatment of cocaine dependence (Gonzalez et al. 2007; Winhusen et al. 2005, 2007). In 2005, a black-box warning was added to the label for tiagabine, warning physicians of new-onset seizures that were noted in some patients (without a history of epilepsy) taking the medication (Gabatril^®^ package insert).

##### Modafinil

Of particular interest in the treatment of cocaine dependence is modafinil because it has a mild stimulant-like effect and produces a mild euphoria (the exact nature of which has been debated in the literature). The drug appears to blunt the desirable effects and the craving associated with cocaine use and reduces cocaine use in a human laboratory setting, with little or no abuse potential (Hart et al. 2007; O’Brien et al. 2006).

#### Naltrexone

Most of the findings concerning the effects of naltrexone on cocaine dependence come from studies of patients with co-occurring alcohol and cocaine dependence (see below), although one study (Schmitz et al. 2001) evaluated its use in patients with only cocaine dependence. This study evaluated the effects of naltrexone in a prospective, randomized trial that compared four groups receiving placebo or naltrexone (50 mg) combined with either relapse prevention therapy or standard drug counseling. The group that received naltrexone combined with relapse prevention treatment outperformed the other three groups, supporting its use, but only in combination with that specific psychosocial intervention.

##### Baclofen

Some evidence of efficacy in treating cocaine dependence also has been shown with the use of baclofen (Shoptaw et al. 2003).

##### Other Medications

Antipsychotic medications do not appear useful for treating cocaine dependence (Amato et al. 2007). A vaccine that causes patients to develop antibodies to cocaine in the blood is in development and has shown promise in treating cocaine dependence (Martell et al. 2005).

#### Opioid Dependence

Opioid dependence in the United States is most often treated with behavioral treatment approaches combined with maintenance therapy with the opioid agonists methadone or buprenorphine. Considerable evidence supports the efficacy of these agents for opioid dependence treatment (Barnett et al. 2001; Connock et al. 2007; Johansson et al. 2007; Mattick et al. 2003, 2004; Van den Brink and Haasen 2006). Although some patients are able to maintain abstinence from opioids following detoxification through involvement in self-help groups such as Narcotics Anonymous, many patients require treatment with opioid agonist medications, and some require maintenance treatment indefinitely. Methadone treatment of opioid dependence is available only through licensed methadone programs that monitor patients’ drug and alcohol use and sometimes provide treatment for co-occurring psychiatric disorders.

Opioid antagonists, including naltrexone (which is FDA approved for this indication), also can be used to treat opioid dependence in patients who are able to transition from physiologic dependence to abstinence. To avoid precipitating acute withdrawal, a patient should be free of opioid use for a minimum of 7 days before being treated with an opioid antagonist (Nunes et al. 2006). In a recent meta-analysis of naltrexone treatment of opioid dependence (Johansson et al. 2006), retention in treatment was a significant moderator of a beneficial effect of the drug. This likely reflects the key problem of adherence to naltrexone that is seen in treatment populations. However, behavioral therapies such as contingency management appear to improve naltrexone adherence and treatment retention in this population (Nunes et al. 2006). Patients should be warned that death by overdose can occur despite opioid receptor blockade by naltrexone or after recent treatment with naltrexone (Gibson and Degenhardt 2007; Gibson et al. 2007).

Baclofen, which has shown some promise in the treatment of alcohol and cocaine dependence (see above), also has been evaluated as a maintenance treatment for opioid dependence (Assadi et al. 2003). In a 12-week, randomized, double-blind, placebo-controlled trial, baclofen was superior to placebo on treatment retention, opioid withdrawal symptoms, and depressive symptoms. However, there was no significant difference in terms of opioid use, alcohol use, opioid craving, or side effects.

#### Cannabis Dependence

There are no medications that have demonstrated efficacy for the treatment of cannabis dependence (Nordstrom and Levin 2007).

## Treatment of AODUDs

As reviewed above, most studies have focused on the treatment of individual SUDs rather than co-occurring disorders. Although studies conducted specifically in AODUD populations are limited, the evidence to date suggests that approaches similar to those used to treat the individual SUDs may be effective. As with the treatment of dependence on individual substances, behavioral therapies provide the “backbone” or main component of treatment for patients with AODUDs. In addition, because the studies evaluating pharmacotherapies in AODUD patients almost always include at least one behavioral therapy component, this review does not examine these types of therapy separately. Table 4 summarizes the evidence for various individual treatments for AODUDs.

Contingency management has been shown to increase treatment retention and improve outcomes across a spectrum of addictive disorders, irrespective of psychiatric severity. Hence, contingency management may serve effectively as a platform for the treatment of AODUDs (Weinstock et al. 2007). A review of treatments for alcoholic methadone patients suggested that making methadone treatment contingent on disulfiram ingestion may effectively reduce drinking and alcohol-related adverse outcomes (Bickel et al. 1987). Along similar lines, contingency management using both prizes and vouchers has been shown to be beneficial for co-occurring opioid and cocaine/stimulant use disorders, as well as other co-occuring substance dependence disorders, including alcohol dependence (Peirce et al. 2006; Petry et al. 2000; Petry and Martin 2002; Prendergast et al. 2006).

### Co-Occurring Alcohol and Cocaine Dependence

Similarities in the pathophysiology of cocaine and alcohol dependence suggest that these disorders may respond to the same medications (Johnson 2005). Carroll and colleagues (1998) examined the effects of disulfiram, CBT, and 12-step facilitation (TSF) on drinking and cocaine use in patients with these co-occurring disorders. Compared with no medication, disulfiram was efficacious in reducing the use of both drugs, and both CBT and TSF were superior to supportive counseling. In a 1-year follow-up, the effects of disulfiram on cocaine use persisted, but no effects were found on drinking behavior (Carroll et al. 2000). Other studies (Carroll et al. 2004; Nich et al. 2004) of disulfiram in cocaine-dependent subjects have shown that co-occurring alcohol use and dependence, as well as female gender, may actually limit the efficacy of the medication.

Hersh and colleagues (1998) found no advantage for naltrexone (50 mg/day) over placebo in the treatment of people with co-occurring cocaine and alcohol use disorders. In a randomized, controlled trial in which alcohol- and cocaine-dependent subjects received either naltrexone (50 mg/day) or placebo, combined with either relapse prevention or drug counseling psychotherapy, Schmitz and colleagues (2004) also found no effects of the medication. In a small, open-label trial of oral naltrexone (150 mg/day), Oslin and colleagues (1999) found that patients with co-occurring alcohol and cocaine dependence reduced both their drinking and their cocaine use. A follow-up, randomized, placebo-controlled trial of a 150-mg daily oral dose of naltrexone, in which patients were stratified by gender, showed that the active treatment reduced cocaine and alcohol consumption among men but not among women (Pettinati et al. 2008*a*). These findings suggest that, at least in men, a higher dose of naltrexone may be efficacious in treating patients with co-occurring cocaine and alcohol dependence.

In a recent, 11-week comparison of disulfiram, naltrexone, or a combination of the two for the treatment of cocaine and alcohol dependence, study participants receiving disulfiram (alone or in combination) had higher rates of combined abstinence (to both alcohol and cocaine) than those on placebo (Pettinati et al. 2008*b*). The combination of disulfiram and naltrexone was superior to either drug given alone, or placebo, on a secondary outcome measure (i.e., the achievement of 3 weeks of continuous abstinence). All the patients received concurrent CBT as a behavioral regimen. A study of 12 patients with co-occurring alcohol and cocaine dependence showed significant reductions in the use of both substances with the addition of naltrexone (50 mg/day) or disulfiram (400 mg/day) to CBT, compared with CBT alone (Grassi et al. 2007).

Because topiramate (at a target dose of 300 mg/day) has demonstrated efficacy in studies of alcohol dependence (Johnson et al. 2003, 2007) and preliminary evidence of efficacy in studies of cocaine dependence (at a target dose of 200 mg/day) (Kampman et al. 2004), it may be an ideal candidate for the treatment of these disorders when they co-occur. Further, topiramate can be safely prescribed to patients taking buprenorphine or methadone, so it could be combined with other treatments for alcohol and drug dependence.

Although modafinil appears to be effective for reducing cocaine use in cocaine-dependent patients, a recent study showed that those with co-occurring alcohol dependence may not experience this benefit (Karila et al. 2007; Vocci and Elkashef 2005). Because alcohol use disorders may have a negative effect on outcomes for cocaine abuse treatment, particularly when patients continue to drink after initiating cocaine abuse treatment, addressing the use of both substances through combined treatment approaches may be optimal (Poling et al. 2007).

### Co-Occurring Opioid and Cocaine Dependence

Buprenorphine appears dose-dependently to reduce cocaine use in opioid-dependent patients, although it does not appear to be any more effective than methadone for that purpose (Montoya et al. 2004, Oliveto et al. 1994; Schottenfeld et al. 1993, 1997). The addition of desipramine to buprenorphine or methadone may augment or facilitate abstinence from cocaine and opioids in patients dependent on both substances (Kosten et al. 2003, 2005; Oliveto et al. 1999). Interestingly, in a placebo-controlled study comparing desipramine alone or in combination with contingency management in buprenorphine-maintained patients, the combined group did significantly better than the other groups on measures of cocaine use (Kosten et al. 2003). High-dose tiagabine (24 mg/day), in addition to methadone, also was superior to placebo in reducing cocaine use in patients dependent on both opioids and cocaine (Gonzalez et al. 2003, 2007). A double-blind, randomized, placebo-controlled trial of disulfiram in methadone-maintained patients with co-occurring dependence on cocaine found that disulfiram significantly decreased cocaine use in these patients (Petrakis et al. 2000). George and colleagues (2000) also found disulfiram to reduce cocaine use and increase the number of weeks of cocaine abstinence in buprenorphine-maintained patients with co-occurring cocaine dependence.

Two randomized controlled studies of bupropion in methadone-maintained patients have shown a possible role for the medication in the treatment of cocaine dependence. Margolin and colleagues (1995) compared bupropion with placebo in a 12-week study of 149 methadone-maintained patients with cocaine dependence. Although there was no overall difference in cocaine use between the two groups, a subgroup of patients that was depressed at the beginning of the study showed a significant reduction in cocaine use. Poling and colleagues (2006) conducted a 6-month trial of bupropion and contingency management in methadone-maintained patients with cocaine dependence. These investigators found that bupropion combined with contingency management reduced cocaine use. Although in this population bupropion without contingency management has not been shown to be efficacious, combining the medication with contingency management may have a synergistic effect. No serious adverse events were reported in either study. However, as with cocaine, bupropion can lower the seizure threshold.

### Co-Occurring Opioid and Alcohol Dependence

Preliminary research suggests that adequate dosing of methadone during maintenance treatment (achieved by increasing the dose until urine screens are negative for all opioids and benzodiazepines) may be effective in reducing both drug and alcohol use in patients with a co-occurring alcohol use disorder (Maremmani et al. 2007). As mentioned above, disulfiram appears to be efficacious in reducing drinking in this population but probably only when continued methadone maintenance is made contingent on disulfiram ingestion (Bickel et al. 1987).

Naltrexone, in both the oral and long-acting injectable forms, has been shown to be effective in treating detoxified opioid-dependent patients and thus can be considered for use in highly motivated individuals (Comer et al. 2006; Johansson et al. 2006; Nunes et al. 2006). However, the high cost of the only long-acting naltrexone formulation available in the United States, combined with reports of patient deaths from opioid overdose stemming from efforts to overcome the blockade produced by naltrexone, require that practical and ethical concerns be evaluated before this approach can be recommended widely to treat opioid dependence (Gibson and Degenhardt 2007; Gibson et al. 2007). Nonetheless, the use of naltrexone for selected patients (e.g., physicians) with opioid dependence appears justified. The use of naltrexone therapy in patients with alcohol dependence and a less severe form of co-occurring opioid use disorder (i.e., abuse rather than dependence, with no history of intravenous use) also may be a viable option because it does not present as great a risk of opioid overdose as may be present in patients with moderate to severe opioid dependence.

## Treatment Recommendations for Patients With AODUDs

For patients with alcohol and cocaine dependence, disulfiram has better empirical support than any other medication. Less compelling evidence exists for the use of either naltrexone or topiramate, but these also should be considered for treatment of these co-occurring disorders. A daily dose of more than 50 mg of naltrexone is needed to treat these disorders but may not be efficacious for women with co-occurring alcohol and cocaine dependence. Topiramate has not yet been studied in AODUD patients, but its safety and efficacy have been demonstrated in patients with alcohol or cocaine dependence. Although the optimal dosage has not yet been determined, preliminary findings suggest that 200 to 300 mg per day, increased gradually over 6 to 8 weeks, is required. Second-line therapies may be effective in patients with cocaine dependence but not alcohol dependence (i.e., modafinil and tiagabine) or vice versa (i.e., acamprosate). Baclofen also could be considered for use in select patients based on evidence of its efficacy in alcohol or cocaine dependence. In addition to an absence of data on the efficacy of these medications in co-occurring cocaine and alcohol dependence, it is unclear whether these medications should be used alone or combined with first-line or other second-line agents. There is limited evidence to support combining disulfiram and naltrexone to treat co-occurring alcohol and cocaine dependence; further research on the combination is needed before it can be recommended as offering an advantage over the use of either medication alone.

For alcohol-dependent patients on methadone maintenance, optimizing the dosage of methadone may help to reduce drinking. Stabilizing opioid withdrawal symptoms and illicit opioid use with methadone or buprenorphine in qualifying patients is an appropriate first step. For patients who continue to drink while receiving opioid maintenance therapy, the first-line alcohol treatments (with the exception of naltrexone) should be considered. Disulfiram may be the medication of choice for such patients, because they also may have a cocaine use disorder. To increase adherence to disulfiram treatment in this patient population, it is probably necessary to require that the patient submit to observed disulfiram ingestion as a condition of continued opioid agonist treatment. Topiramate may be an option in this population as well, though there are no published reports to guide this approach.

For patients dependent on opioids and cocaine, adequate dosing of buprenorphine and methadone may reduce use of both substances. This approach should be considered as a first-line therapy for such patients, as long as the severity of their opioid dependence warrants opioid agonist therapy. If monotherapy with buprenorphine or methadone is inadequate to control co-occurring cocaine use, adding disulfiram to either agonist treatment should be considered. Research also suggests that adding desipramine or high-dose tiagabine to an opioid agonist maintenance regimen can be helpful. However, caution is necessary in prescribing medications to patients with co-occurring opioid and cocaine dependence as they may have significant medical co-morbidity, including cardiac and liver disease. This strategy also has the potential to cause drug–drug interactions. As is true for the treatment of patients with other combinations of AODUDs, pharmacotherapy in this patient group should be combined with behavioral therapies, such as contingency management, which may be particularly useful in combination with desipramine.

## Summary of General Treatment Recommendations

Although the treatment literature is rapidly growing for individual SUDs, there is a paucity of systematic research on treatments for AODUDs. The existing literature shows that, as with DUDs alone, combined behavioral and psychopharmacological treatments for patients with AODUDs are likely to be optimal. At a minimum, patients should be encouraged to participate in a 12-step program and are likely to benefit from the addition of group or individual therapies that use motivational enhancement and cognitive-behavioral techniques. When available, the use of contingency management is likely to enhance outcomes for patients with AODUDs. The use of medications to improve outcomes in AODUD patients has shown initial promise, particularly for co-occurring alcohol and cocaine dependence.

Treatment planning for patients with AODUDs should include medical and psychiatric evaluations and integrated treatment to address co-occurring substance use and psychiatric disorders. Given the burden of psychopathology, patients with AODUDs often may require a higher level of care (e.g., inpatient rehabilitation, psychiatric partial hospital or intensive out-patient “dual diagnosis” programs) for initial stabilization. Medications with beneficial effects on drinking behavior and other drug use should be used in combination with behavioral interventions. In short, treatment for patients with AODUDs should start with a motivational intervention, with a focus on developing a therapeutic alliance. In these efforts, the clinician should be mindful of the patient’s stage of change and level of motivation, utilize empathic listening and expression, address the patient’s goals and needs, emphasize and promote self-regulation skills, utilize multiple treatment modalities, actively address co-occurring medical and psychiatric illness, and promote adherence to the treatment program.

## Figures and Tables

**Table 1:** Summary of Research on Behavioral Therapies for Specific Substance Use Disorders

	**Alcohol**	**Cocaine**	Opioid*	**Marijuana**
CBT	++ (A)	++ (A)		+ (B)
MET	++ (A)	+/− (B)	+/− (B)	+ (B)
CM	++ (A)	+ (A)	+ (A)	+ (B)
BI	++ (A)			
TSF	+ (A)	+/− (B)	+/− (B)	
CET	+ (B)			
BCT	+ (B)			
CRA	++ (A)			

NOTES: BCT = Brief Couples Therapy, BI = Brief Intervention, CBT = Cognitive-Behavioral Therapy, CET = Cue Exposure Therapy, CM = Contingency Management, CRA = Community Reinforcement Approach, MET = Motivational Enhancement Therapy, TSF= Twelve-Step Facilitation.

For level of evidence supporting the use of therapies: (−) indicates that the treatment appears not to be efficacious, (+/−) indicates conflicting results or preliminary evidence of efficacy, (+) indicates evidence of efficacy from randomized controlled trials, and (++) indicates evidence of efficacy from multiple trials and/or meta-analyses.

Evidence-based strength of recommendation taxonomy (SORT; as defined by Ebell et al. 2004): (A) Recommendation based on consistent and good-quality patient-oriented evidence. (B) Recommendation based on inconsistent or limited-quality patient-oriented evidence. (C) Recommendation based on consensus, usual practice, opinion, disease-oriented evidence, or case series for studies of diagnosis, treatment, prevention, or screening.

* Behavioral treatments for opioid dependence likely are most effective when combined with pharmacotherapy.

**Table 2:** Summary of Research on Pharmacotherapies for Specific Substance Use Disorders

	**Alcohol**	**Cocaine**	Opioid*
Disulfiram	+/−* (B)	+ (B)	
Naltrexone	++ (A)	+/− (B)	+/−* (B)
Acamprosate	++ (A)		
Topiramate	+ (A)	+/− (B)	
Ondansetron	+/−** (C)	+/− (C)	
Sertraline/SSRI	+/−** (C)	− (C)	
Carbamazepine	+ (B)	− (C)	
Valproate	+/− (B)	+/− (B)	
Tiagabine		+/− (B)	
Aripiprazole	− (C)		
Modafinil		+ (B)	
Quetiapine	+/−** (C)		
Olanzapine	− (C)	− (C)	
Lithium	− (C)		
Baclofen	+/− (B)	+/− (B)	− (C)
Buprenorphine			++ (A)
Methadone			++ (A)

NOTES: For level of evidence supporting the use of therapies: (–) indicates that the treatment appears to be ineffective, (+/–) indicates either conflicting results or preliminary/potential evidence of efficacy, (+) indicates support from randomized controlled trials, (++) indicates support for efficacy from multiple trials and/or meta-analyses.

* Effective in highly motivated patients that will adhere.

** May be effective in certain subtypes of alcohol dependence, or in dually-diagnosed individuals.

Evidence-based strength of recommendation taxonomy (SORT; as defined by Ebell et al., 2004): (A) Recommendation based on consistent and good-quality patient-oriented evidence. (B) Recommendation based on inconsistent or limited-quality patient-oriented evidence. (C) Recommendation based on consensus, usual practice, opinion, disease-oriented evidence, or case series for studies of diagnosis, treatment, prevention, or screening.

**Table 3:** Mechanisms of Action of Medications Used to Treat Alcohol and Other Drug Use Disorders

**Name**	**Main Mechanism**	**Pharmacologic Action**
**Disulfiram**	Aversive therapy: consumption of alcohol within up to 2 weeks of ingesting the medication produces the disulfiram–ethanol reaction, consisting of flushing, palpitations, increased heart rate, decreased blood pressure, nausea/vomiting, sweating, dizziness, among other possible symptoms. Anticipating these effects averts drinking. Also may diminish the “high” produced by cocaine, reducing cocaine craving. It also directly reduces cocaine use independent of the patient’s level of drinking.	For alcohol dependence: inhibits the action of the metabolic enzyme aldehyde dehydrogenase, resulting in a buildup of acetaldehyde during ethanol metabolism. May also indirectly modulate receptors for the neurotransmitter glutamate. For cocaine dependence: inhibits the function of the enzyme dopamine β-hydroxylase, thus inhibiting metabolism of the neurotransmitter dopamine, thereby increasing the unpleasant effects of cocaine. Also increases cocaine plasma concentrations.
**Naltrexone**	Attenuates the rewarding effects of alcohol in the brain and also may reduce the conditioned anticipation of those effects, as manifested in the urge to drink.	Is an opioid receptor antagonist; it blocks the effects of increased endogenous opioids (caused by alcohol) on dopaminergic transmission in the nucleus accumbens.
**Acamprosate**	Postulated to reduce relapse risk by reducing the urge to drink and the drive to experience the negative reinforcing effects of alcohol.	Essentially a modulator of glutamatergic transmission; has a complex mechanism of action that involves the modulation of certain glutamate (NMDA) receptors, calcium channels, and other downstream intracellular molecular events.
**Topiramate**	Postulated to reduce the rewarding effects of drinking and the conditioned anticipation of those effects, as manifested in the urge to drink.	Multiple pharmacologic effects; facilitates some chemical connections in the brain and inhibits others. Indirectly attenuates dopaminergic transmission in the nucleus accumbens in response to drinking and directly attenuates neuronal glutamatergic hyperexcitability in the absence of alcohol. Mechanism in reducing cocaine use is unknown but is perhaps related to its GABAergic and glutamatergic effects.
**Modafinil**	Attenuates the rewarding effects of cocaine, perhaps reducing the urge to use as well. Acts as a “functional partial agonist,” meaning that it has stimulant-like effects but also reduces cocaine self-administration and euphoria.	Unknown mechanism of action; thought to have minimal abuse potential and limited euphoric effects.
**Buprenorphine**	Blocks the rewarding effects of opioids, reduces withdrawal and the urge to use the drugs.	Partial agonist at μ-opioid receptors, blocks κ-opioid receptors. Unknown mechanism in reducing cocaine use in opioid-dependent patients.
**Methadone**	Blocks the rewarding effects of opioids, reduces withdrawal and urge to use the drugs.	Agonist at μ-opioid receptors; has unique pharmacokinetic and pharmacodynamic properties, including a long latency to peak blood concentrations (thereby minimizing its euphoric effects), a long half-life (reducing acute withdrawal symptoms), and opioid cross-tolerance (reducing euphoria from heroin and other short-acting opioids). Unknown mechanism in reducing cocaine use in opioid-dependent patients.

NOTE: An agonist is a substance that binds to a specific receptor and triggers a response in the cell. For references see text, as well as Kranzler and Ciraulo 2005.

**Table 4:** Summary of Research on Treatments for AODUDs

	**Alcohol/Cocaine**	**Alcohol/Opioid**	**Cocaine/Opioid**
Disulfiram	+/− (B)	+/− (B)**	+ (B)
Naltrexone	+/− (B)	* (C)	* (C)
Buprenorphine			+ (B)
Methadone		+/− (C)	+ (B)
Desipramine			+ (B)
Topiramate	* (C)		
Baclofen	* (C)		
Tiagabine			+/− (B)
CBT	+ (B)		+ (B)
CM	+/− (B)	+ (B)	+ (B)
TSF	+/− (B)		

NOTES: AODUDs = Alcohol and other drug use disorders, CBT = cognitive-behavioral therapy, CM = contingency management, TSF = twelve-step facilitation.

* = recommendation synthesized from studies performed in primarily alcohol or cocaine dependent subjects, not specifically in the dually dependent group

** = may only be effective when continued opioid agonist therapy is made contingent on disulfiram ingestion.

For level of evidence supporting the use of therapies: (−) indicates that the treatment appears to be ineffective, (+/−) indicates conflicting results or preliminary evidence of efficacy, (+) indicates evidence of efficacy from randomized controlled trials, (++) indicates evidence of efficacy from multiple trials and/or meta-analyses.

Evidence-based strength of recommendation taxonomy (SORT; as defined by Ebell et al., 2004): (A) Recommendation based on consistent and good-quality patient-oriented evidence. (B) Recommendation based on inconsistent or limited-quality patient-oriented evidence. (C) Recommendation based on consensus, usual practice, opinion, disease-oriented evidence, or case series for studies of diagnosis, treatment, prevention, or screening.

## Footnotes

1 In opioid agonist therapy, patients receive a drug, such as methadone or buprenorphine, that is chemically similar to opioid drugs.

2 Contingency management is the systematic reinforcement of desired behaviors and the withholding of reinforcement or punishment of undesired behaviors.

3 Receptor antagonists block the binding of other substances (i.e., agonists) that trigger responses in the cell.

4 An intention-to-treat analysis is an analysis based on the initial treatment intent not on the treatment eventually administered. It is based on the assumption that, as in real life, sometimes patients do not all receive optimal treatment, even though that was the initial intention.
